# Characterizing International Travel Behavior from Geotagged Photos: A Case Study of Flickr

**DOI:** 10.1371/journal.pone.0154885

**Published:** 2016-05-09

**Authors:** Yihong Yuan, Monica Medel

**Affiliations:** Department of Geography, Texas State University, San Marcos, Texas, 78666, United States of America; Beijing University of Posts and Telecommunications, CHINA

## Abstract

Recent advances in multimedia and mobile technologies have facilitated large volumes of travel photos to be created and shared online. Although previous studies have utilized geotagged photos to model travel patterns at individual locations, there is limited research on how these datasets can model international travel behavior and inter-country travel flows—a crucial indicator to quantify the interactions between countries in tourism economics. Realizing the necessity to investigate the potential of geotagged photos in tourism geography, this research investigates international travel patterns from two perspectives: 1) We apply a series of indicators (radius of gyration (ROG), number of countries visited, and entropy) to measure the descriptive characteristics of international travel in different countries; 2) By constructing a gravity model of trade, we investigate how distance decay influences the magnitude of international travel flow between geographic entities, and whether (or how much) the popularity of a given destination (defined as the percentage of tourist income in national gross domestic product (GDP)) affects travel choices in different countries. The results provide valuable input to various commercial applications such as individual travel planning and destination suggestions.

## 1. Introduction

Recent studies have investigated the usage of big data to generalize, model, and predict human mobility and travel behavior, including location-based social media (LBSM) [1, 2], mobile phone tracking [3, 4], Global Positioning System (GPS) logs, or a combination of the above [5]. Among these new big data sources, the usage of LBSM in modeling travel behavior has grown rapidly: these data are user-generated, geo-located, and contain varying types of contextual information (text, videos, images, etc.), therefore can be potential resources to characterize activities’ patterns in various temporal scales–from daily to yearly–and users’ social perceptions of place [6].

Specifically, researchers have explored the potential of employing geotagged photos to analyze individual and aggregated travel behaviors [7–9]. Recent advances in multimedia and mobile technologies have facilitated large volumes of travel photos to be created and shared online. Unlike traditional travel surveys or actively collected GPS logs (e.g., in human-participant experiments), these datasets often cover a large sample size and can easily be accessed through crowd-sourcing toolkits [8]. Hence, geotagged photos often provide information or solutions faster and in greater detail than traditional means for obtaining the spatio-temporal footprint of travelers [10]. Although previous studies have investigated utilizing geotagged photos to model travel patterns at individual locations (e.g., the study on Hong Kong tourists in [8]), as well as predicting individual travel behavior and providing future destination recommendations [11], there is limited research on how these datasets can model international travel behaviors and inter-country travel flows—a crucial indicator to measure the interactions between countries and model international capital flows in tourism economics [12].

Realizing the necessity to investigate the potential of geotagged photos in modeling inter-country travel behavior, this research aims to investigate international traveling from two perspectives: 1) we apply a series of indicators (radius of gyration (ROG), number of countries visited, and entropy) to measure the descriptive characteristics of international traveling in different countries. These three indicators measure both the “morphology” (e.g., the scale) of traveling and the internal structure of how travel interest distributes in different countries (e.g., do users in the United States (U.S.) upload similar number of photos in each visited country, or is the photo uploading activity less evenly distributed?); 2) the spatial decay effect has been a continuing topic in many research fields such as immigration, transportation, and international tourist studies (e.g., the decay of interaction flows between locations) [13]. A thorough understanding of these behavior patterns is crucial for promoting the development of the tourism industry and maintaining sustainable mobility. Hence, in this research, we also investigate how distance decay influences the magnitude of international traveling between geographic entities, and whether (or how much) the popularity of a certain destination (defined as the percentage of tourist income in national gross domestic product (GDP) of a certain country) affects travel choices in different countries. Among all potential models, we chose the gravity model of trade due to its effectiveness in predicting the degree of interaction, simplicity of equation, and its ability to deal with flows in both directions [14]. This study contributes to the field from the following perspectives: First, empirically, we analyze three aspects of international travel pattern (ROG, number of countries visited, and entropy), as well as how these patterns correlate with the spatial distance and the socio-economic factors of a certain country. Although similar studies have been conducted based on LBSM, such studies mainly focus on travel distances, and there has not been sufficient study on how to explore various aspects of international travel behaviors from user-generated geotagged photos. Second, methodologically, we demonstrate the effectiveness of employing a variation of the gravity model of trade in international traveling, where the travel behavior is bilateral.

This paper is organized as follows: Section 2 describes related studies in the areas of travel behavior, LBSM, and the application of gravity models. Section 3 illustrates the fundamental research design, including the Flickr dataset and the methodology. Section 4 presents the data analyses and results, and discusses various aspects of the output in detail. We conclude this research and present directions for future work in Section 5.

## 2. Related Studies

### 2.1. Location-Based Social Media, Human Mobility, and Tourism Geography

The continued development of social networking sites (SNS) like Twitter, Facebook, and Flickr provides ever-increasing opportunities to explore mobility patterns of individuals in diverse geographic environments, social statuses, and cultural backgrounds. Meanwhile, the widespread use of smart phones, which are equipped with sensors that allow users to instantly locate themselves, has brought another crucial aspect to this development: location. Researchers have defined location-based social media as “Social Network Sites that include location information” [2]. Despite potential issues such as low sampling resolution, previous studies have demonstrated the effectiveness of LBSM data to analyze human movement and to construct more powerful mobility models [5, 15]. For instance, Gao et al. [16] have investigated the role of social correlation in users’ check-in behavior to improve the accuracy for location prediction. Another study by Hasan et al. [17] analyzed the timing distribution of visiting different places depending on activity category for individual users.

In addition to the analysis of individual activity patterns and space, LBSM data provides a great opportunity to investigate how human beings’ mobility is shaped by urban environments and how the latter may be managed or designed to better suit the needs of the former. Previous studies have also attempted to classify neighborhoods (such as the Livehoods project [18]) and/or extract activity anchor points (e.g., “home” and “work”) [19] from LBSM. A number of studies used check-in data from Foursquare to analyze how the population’s spatio-temporal activities are defined by or reflect the spatial structure of the particular cities they are in [18, 20].

On the other hand, the impact of LBSM on tourism geography has raised worldwide interests among scholars. Researchers define tourism geography as the study of travel and tourism, which includes a wide range of social and cultural activities, such as the sociology and management of tourism [21]. A series of studies have focused on the “location” component in SNS (i.e., the usage of LBSM and its indication of travel behavior patterns at varying spatio-temporal scales). Previous studies have concentrated on individual-level trajectory analysis and location prediction, such as identifying future travel destinations based on LSBM check-in data [22, 23]. Other research, however, focused more on aggregated patterns (e.g., domestic or international travel flows) in specific tourist sites [8]. Traditionally, international travel flows are often measured by an economic index (e.g., tourist income) or airline transportation data. These data are often static, posted by authorities with a time-lag, and with limited information to calculate movement indicators for each individual; therefore, they cannot fully reflect the dynamic nature of international traveling in the current mobile society as massive datasets from LBSM [24].

In 2014, Yahoo published the Flickr Creative Commons 100M dataset containing one hundred million photos [25]. Since then, researchers in communication, computer science, geography, and related fields have been utilizing this open dataset to analyze human behavior from various perspectives. For example, researchers have tested several new image retrieval and information searching algorithms based on this dataset [26, 27]. In the geographic information science (GIS) field, a few studies have utilized this dataset to analyze individual mobility patterns, such as identifying users’ points of interest (e.g., home locations) [28] or extracting spatio-temporal keywords for moving objects [29]. However, aggregated-level mobility analysis based on this dataset is still limited, especially at the international level. Sun et al. [7] analyzed Flickr data to reveal the spatial distribution of tourist accommodation in one particular city (Vienna) through different seasons. A recent work by Beiró et al. [30] proposed a hybrid model to predict human mobility flows based on the classical gravity model, under a stacked regression procedure. However, their work focuses on comparing domestic travel flows in the U.S. with airline transportation networks instead of modeling international travel flows. Barchiesi et al. [31] analyzed the travel flows to the United Kingdom (U.K.). However, this study mainly concentrated on validating the magnitude of travel flows with authority data for one country. There has not been sufficient study to extract the tourism interactions between countries from geotagged photos at a global level, and this research aims to provide an empirical study from this perspective.

### 2.2 Spatial Interaction and the Gravity Model of Trade

As discussed in Section 1, spatial interaction is a continuing topic in many research fields including transportation (e.g., traffic flows between locations) and immigration (e.g., immigrant flows between countries) [13, 32, 33]. Researchers have employed different models to investigate how distance decay may influence the magnitude of interactions between geographic units. Among all potential models, the gravity model is commonly-used due to its effectiveness in predicting the degree of interaction, its simplicity of equation, and its ability to deal with flows in both directions [13, 14]. For instance, Hardy et al. (2012) investigated how gravity models can help determine the role of distance in volunteered geographic information (VGI) production, and Liu et al. (2014b) used them to explore relatedness between Chinese provinces.

The traditional gravity model is defined as:

$$I_{ij}=K\frac{P_{i}P_{j}}{D_{ij}^{\beta }}$$ (1)

where *P*_*i*_ and *P*_*j*_ are the “conceptual sizes” (relative importance) of two countries *i* and *j* in a certain topic, *D*_*ij*_ represents the distance between them, and *I*_*ij*_ denotes the interaction/connection between *i* and *j*. *β* (the distance friction coefficient) shows the degree of distance decay—larger *β* indicates a higher degree of distance decay. *K* is a constant to adjust the magnitude of interaction that does not affect the model fitting.

However, researchers have also noticed certain limitations of the traditional gravity model, such as the inability to model bilateral and imbalance interactions between two geographic entities. For example, in international economics, trade flows between countries *I* and *J* are associated with the specific direction of export / import countries (i.e., *I*_ij_ ≠*I*_ji_), therefore the interaction cannot be fully represented in the traditional gravity model. An extended model can be written as:

$$I_{ij}=K\frac{P_{i}^{\beta_{1}}P_{j}^{\beta_{2}}}{D_{ij}^{\beta_{3}}}$$ (2)

where *β*_1_ and *β*_2_ shows how the “conceptual sizes” of two countries (often measured by their GDP) contribute to the interaction term *I*_ij_ [34]. For instance, it is possible that the GDP of China plays a different role in the export of Chinese products to foreign countries and the import of foreign products into China, which can result in different *β*_1_ and *β*_2_ values in Eq (2). Besides international economics, the gravity model of trade is also widely adopted in studies where a bilateral interaction may exist, such as migration and transportation [35, 36]. In this research, we further apply this model to the field of tourism geography to analyze the imbalanced interaction of travel flows between countries. The basic hypothesis is that for travelers from country *A*, both distance and the popularity of the destination can affect their choice of travel destination (measured by the *β* values in Eq 2); however, the influence of these two factors may be bilateral (*β*_A->B_ ≠ *β*_B->A_), where *β*_A->B_ stands for the magnitude of *β* values or residents in country *A*(*B*) traveling to country *B*(*A*). The detailed research design is discussed in Section 3.

## 3. Research Design

### 3.1. Dataset

**Fig 1:**
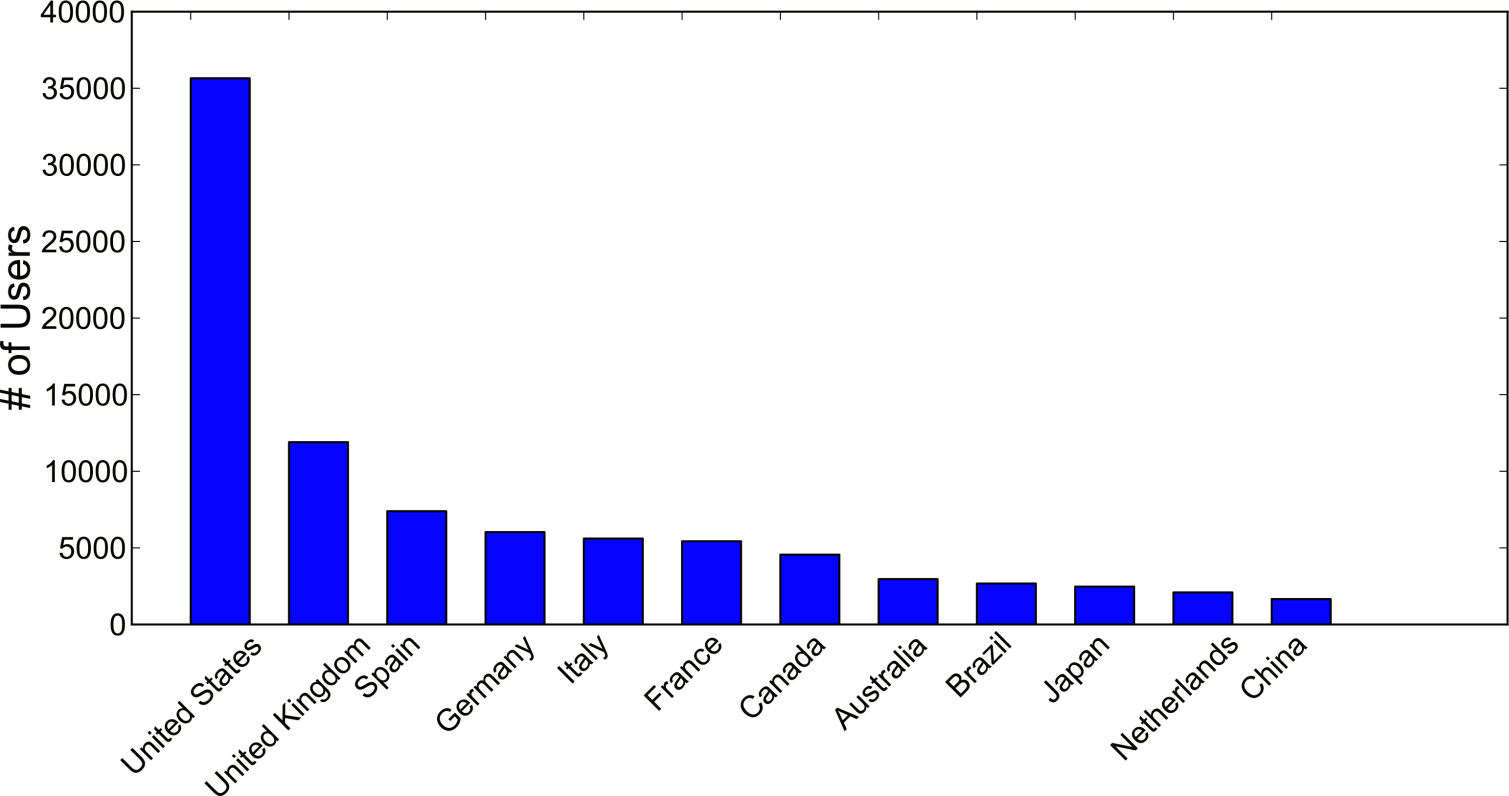
Numbers of Users in Selected Countries.

This research utilizes a publicly available dataset “Creative Commons” published by Flickr in 2014 [37]. This dataset covers 100 million Flickr Images randomly sampled globally during the years 2004–2014, of which approximately 20% are geotagged. The geographic information is extracted from user contributed data, such as the built-in GPS module in smart phones. The total number of unique users is 214,600. The dataset was further reduced by keeping only the users who have uploaded more than 5 photos. We also extracted the residence country from user profile via Flickr API. For users that do not provide their residence country, we use the country where they uploaded the most pictures as a “primary residence country”. We further selected the users who reside in the top 12 countries with the most photos uploaded (Fig 1).

Note that the user group of LBSM is not a randomly selected population. Each social media platform has certain characteristics—things that it allows and makes easy versus things that are difficult to accomplish [38]. This helps to shape behavior as well as the user group (e.g., race, gender, age) on social media sites [39]. Based on a survey on TripAdvisor.com, travel activities are closely connected with online photo sharing, where 55.6% of the survey participants were engaged in posting/sharing photographs [40]. Since the main functionality of Flickr is photo sharing, it better reflects tourist activities than a functionally less specialized SNS such as Twitter. Compared to another geolocation-oriented photo sharing service Panoramio, Flickr has a much larger user base and a faster updating rate. For instance, by November 12^th^ 2015, Panoramio was reported to have 67,121,664 uploaded images, whereas in March 2013, Flickr already had a total of 87 million registered members and more than 3.5 million new images uploaded daily [41, 42].

### 3.2. Methodology

#### 3.2.1. Data preprocessing and defining indicators

As an exploratory analysis, we first calculate several descriptive statistics of the 12 selected countries. For each user we calculate the total number of pictures uploaded, the number of countries where the user posted pictures, the number of images uploaded in each visited country, and the user’s country of residence as defined in Section 3.1. In the case where the resident country is not provided in the user’s profile, we estimated the residence based on where the most number of photos were uploaded. To verify this method, we also conducted a random sampling of 10,000 users who publicly revealed their resident country, which demonstrates that more than 85% of users upload the most photos in their resident country.

In human activity analysis, researchers have applied several approximations and measurements to depict the basic morphology (e.g., size, shape, etc.) of individual activity space. In addition, previous studies also emphasized the reasons why activity space forms (i.e., the internal structure of activity space). Here we adopt a similar framework for international travel [43]. A series of indicators are defined to depict the travel characteristics of Flickr users.

- Radius of Gyration (ROG)–This is defined as an indicator to show the scale of travel. We adopt the equation in González, Hidalgo, and Barabási [44].

$$r_{g}=\sqrt{\frac{1}{n}\sum_{i=1}^{n}{\left(r_{i}-r_{cm}\right)}^{2}}$$ (3)

where *r*_i_ are the *i* = 1, …, *n* positions recorded for a given user, and *r*_cm_ is the mass center of its trajectory. We also eliminated the users whose ROG equals zero, as these users uploaded all pictures from the same geographic coordinates.

- Number of countries visited–This is a common indicator applied in multiple studies to demonstrate the activeness of a traveler [45–47].
- Entropy of pictures uploaded at different countries (except for the identified resident country described earlier in this section). This is an indicator to show how evenly their travel interest is distributed, defined as:

$$E=\text{-}\sum_{i=1}^{N}p_{i}\;\log_{2}p_{i}$$ (4)

where *p*_i_ is the percentage of photos uploaded in country *i* for a given user and *N* stands for the total number of distinct countries visited by a given user (except for the resident country). In mobility studies, entropy usually characterizes the heterogeneity of visitation patterns. For instance, a user who takes more than 50 photos in every country (e.g., an avid traveler or photographer) is more likely to have an equal level of interest in each destination compared to someone who shows a more focused preference (e.g., uploaded more than 200 photos to a certain country (e.g., Italy), but less than 10 photos in any other places he/she visited). These three indicators provide valuable input to understand the generic patterns of international travel behavior in the Flickr dataset.

#### 3.2.2. Fitting the gravity model of trade

As illustrated in Section 2.2, this research employs the gravity model of trade to quantify the interaction between countries based on international traveling. The objective is to identify how outgoing travel flows correlate with the magnitude of “tourism popularity” (defined as the percentage of tourist income in the total GDP of a given country), as well as how this interaction correlates with the distance between the origination and destination countries. Our hypothesis is that the residents of each country may express varying preferences towards “famous tourist sites” and “nearby tourist sites”. In this research we focus on the outgoing flows of each country (i.e., to answer questions like “where do people resident in country *A* travel to?”), so *P*_i,_ *β*_1_ in Eq 2 can be viewed as constant and the model is further modified as:

$$I_{ij}=K\frac{P_{j}^{\beta_{2}}}{D_{ij}^{\beta_{1}}}$$ (5)

where *P*_*j*_ is the “conceptual size” (relative importance, defined as the percentage of tourism income in the total GDP) of the destination country *j*, *D*_*ij*_ represents the distance between countries *i*,*j*, and *I*_*ij*_ denotes the magnitude of interaction between *i* and *j*, defined as the percentage of users from country *i* who have visited country *j* among all users from country *i*. *K* is the same constant as in Eq (1). Coefficient *β*_1_ investigates the potential impact of the distance decay effect. As illustrated in previous studies in human mobility, transportation, and regionalization [44, 48], a higher *β*_1_ value indicates a stronger distance decay effect. Coefficient *β*_2_ indicates how the popularity of the tourism industry in the destination country influences the interaction (e.g., do residents in country *i* prefer to visit closer countries or more popular travel destinations regardless of distance)? Based on the above definitions, we calculate the best fit of coefficient *β*_1_ and *β*_2_ based on a Poisson regression model [49].

The exploratory analysis and the gravity model fitting aim to analyze the international traveling patterns of Flickr users from two differential perspectives: the former focuses on the morphology and magnitude of user activity space in each individual country, whereas the latter focuses on inter-country interactions from a more integral perspective. As argued by Liu et al. [50], the relatedness of two geographic entities can be explored from two perspectives: similarity and connection/interaction. The two analyses in Sections 3.2.1 and 3.2.2 can also be viewed as exemplary studies from these two perspectives. The detailed analyses and model fitting results are illustrated in Section 4.1.

## 4. Analysis, Results, and Discussion

### 4.1. Exploratory Analysis

Table 1 shows the summary statistics of the selected top 12 countries with the most users in the dataset. The three indicators (ROG, # of countries visited, and entropy) for each country are averaged among all residents with more than five photos uploaded in the sample set. As can be seen, the three indicators exhibit distinct patterns among countries.

**Table 1:** Basic Statistics of the 12 Selected Countries.

*Country*	*# of Users*	*# of Photos*	*ROG (km)*	*# of Countries visited*	*Entropy*	*Size of Country (km*^*2*^*)*
***United States***	35653	17722190	1273.992	2.746164	0.529724	9147420
***United Kingdom***	11899	5638358	929.96	3.809144	0.816171	241930
***Spain***	7395	2265700	709.2933	3.496416	0.79686	500010
***Germany***	6030	1729482	729.2576	3.492869	0.756285	348570
***Italy***	5610	1338208	764.2031	3.7041	0.913752	294140
***France***	5433	1772792	925.6718	3.563961	0.852483	547557
***Canada***	4558	1790124	1210.952	3.076349	0.697772	9093510
***Australia***	2963	1100660	2442.972	3.289234	0.722354	7682300
***Brazil***	2673	70705	1056.817	2.567901	0.537113	8358140
***Japan***	2473	1319210	1782.103	3.166599	0.733971	364550
***Netherlands***	2094	936167	837.9643	4.596466	1.023232	33730
***China***	1660	609529	1996	3.624096	0.810386	9388211

**Fig 2:**
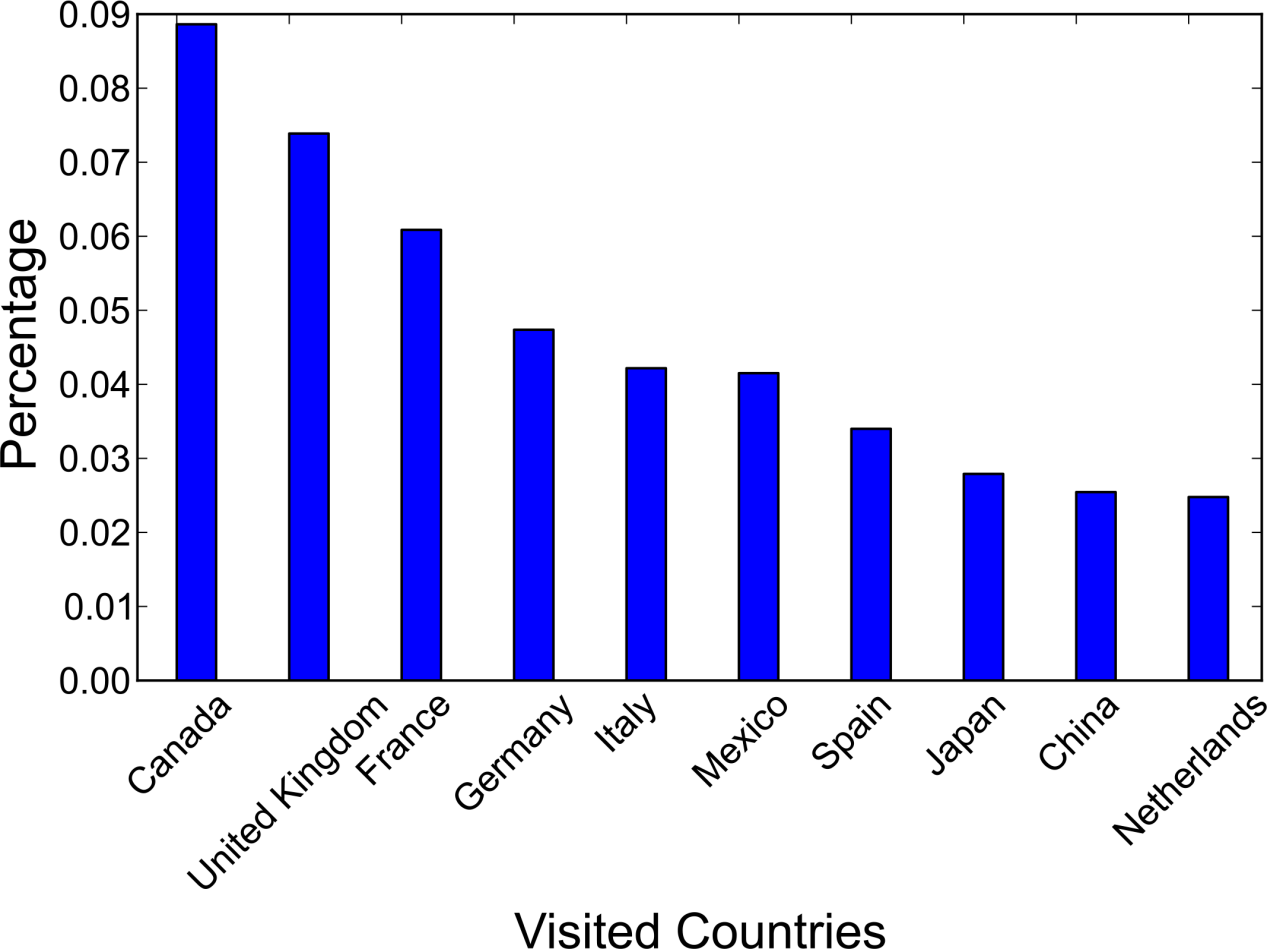
Percentage of U.S. Users Visiting Each Country.

- **ROG**: Countries with a larger ROG mainly locate in Asia (e.g., China and Japan) and Australia, indicating that residents in these countries tend to visit faraway destinations (including both domestic and international travel). Potential reasons include the size of the home country and residents’ general interests for certain travel destinations at the societal level (e.g., European countries are popular vacation destinations for Chinese middle class families [51]), and the lack of international tourism resources from adjacent countries (e.g., Australia is geographically isolated). On the other hand, European countries averagely have a ROG of less than 1000 kilometers, and this is potentially due to the availability of rich tourism resources from nearby countries.
- **Number of Countries visited**: This indicator is relatively stable for the 12 selected countries, with the majority ranging from 3–4. In general European residents visit more countries than North American residents (average 3.7 vs. 2.9). The lowest value appears in the only Southern American country (Brazil) in the sample set, with 2.5 countries visited. This can be considered a general indicator to measure people’s interest in visiting foreign countries (regardless of the distance between countries or preference of specific destinations); however, it cannot reflect the internal structure of visiting patterns.
- **Entropy**: The entropy values also exhibit different patterns among the 12 countries. For instance, Netherlands and Italy have the highest entropy values (1.02 and 0.91 respectively), indicating that the photo uploading from residents in these two countries are more evenly distributed when they travel to a foreign country, whereas the U.S. and Canada indicate a more imbalanced distribution, indicating that the travel interests of these two countries are more focused. Fig 2 shows the visiting patterns for the U.S. as examples. As can be seen, U.S. residents show a strong preference for the top two destinations (Canada and the UK).

Moreover, the size of a country may have a substantial impact on the travel behavior of its residents, for example, residents of a large country may have many domestic destinations to choose from (and therefore require fewer trips abroad). To further explore these patterns, we also calculated the Pearson correlation coefficient between the size of a country and each of the three indicators, as well as the correlation coefficient between each pair of the three indicators (ROG, the number of countries visited, and entropy). As can be seen from Table 2, the number of countries visited is highly correlated with the entropy value. This is conceptually related to the findings in a recent work on Twitter user analysis [52], where the authors identified that tweets tend to distribute more uniformly in cells for larger cities. In this study we found that a larger number of countries visited can be expected to be associated with a more balanced distribution of countries visited and therefore a larger entropy. Future research may choose one of the two indicators to demonstrate the “variety” of international traveling. On the other hand, the size of home countries is positively correlated with ROG, but negatively correlated with the number of countries visited or the entropy value (all with significance level *p*<0.1), indicating that users living in larger countries travel longer to reach their destinations. However, their “variety” of international destinations may be lower due to the fact that larger home countries may offer more domestic tourist resources.

**Table 2:** Correlation coefficients and *p* values.

	ROG (km)	# of Country visited	Entropy	Size of Country (km^2^)
**ROG (km)**	1	0.260(*p* = 0.41)	-0.263(*p* = 0.40)	0.560(*p* = 0.058)
**# of Country visited**	-	1	0.955(*p*<0.01)	-0.617(*p* = 0.032)
**Entropy**	-	-	1	-0.669(*p* = 0.017)
**Size of Country (km**^**2**^**)**	-	-	-	1

**Fig 3:**
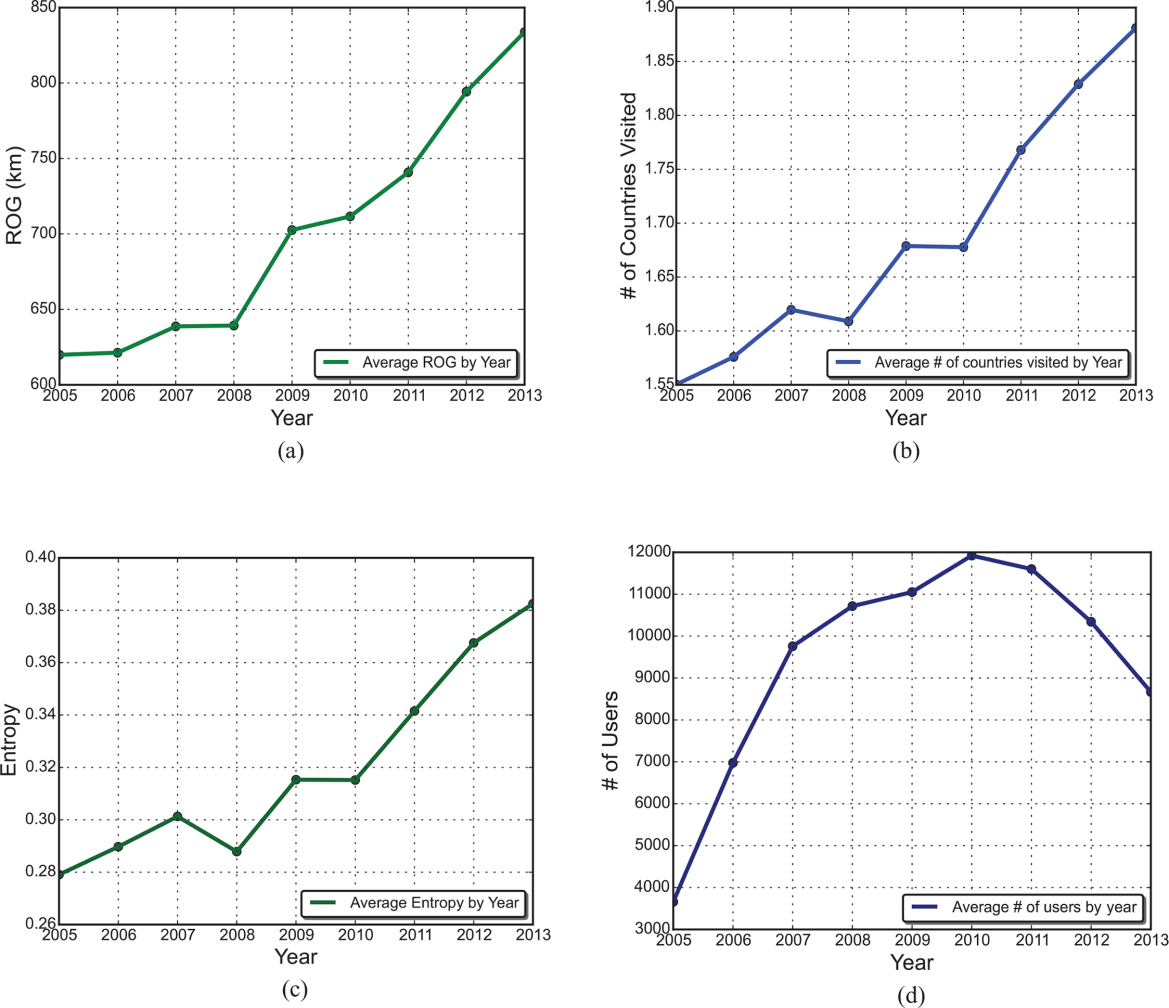
Yearly change of indicators for U.S. users. (a) ROG; (b) number of countries visited; (c) entropy; (d) number of users.

As discussed in Section 2.1, another benefit of applying LBSM data in travel analysis is that these data can easily be collected over years, and therefore they are able to reflect the dynamic nature of international traveling. Fig 3 shows the yearly travel patterns for U.S. users. As can be seen, all three indicators kept increasing from 2005 to 2013. There are several potential reasons that may lead to this pattern. First, it is possible that users are more active on Flickr every year, so the ascending indicators may simply be a result of more sample points; however, as shown in Fig 3D, the number of users started to drop since 2010. Therefore, the upward tendency of indicators partially reflects that U.S. users have a larger activity space and are increasingly involved in international travel in the past few years.

### 4.2. Gravity Model Fitting

**Fig 4:**
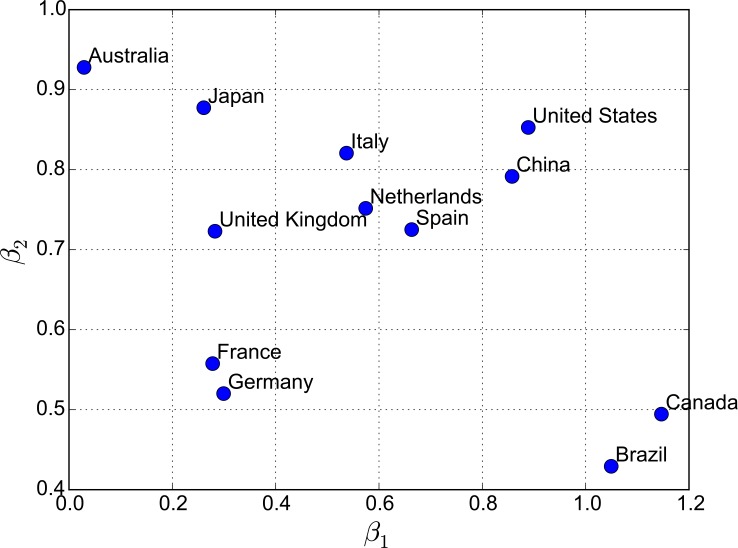
Fitted *β*_1_ and *β*_2_ Values.

As discussed in Section 3, the three defined indicators depict descriptive statistics of travel patterns in each country. However, they fail to address the magnitude of interaction between countries (i.e., travel flow), as well as how the interaction relates to various factors such as the distance. To quantify such interaction, we construct gravity models for each of the 12 countries. These models aim to answer two questions: 1) How does distance decay play a role in international traveling?, and 2) how does the prosperity of tourism markets in a destination country affect people’s travel decisions (i.e., do people in different countries tend to travel to “nearby places” or “popular places”)? Table 3 and Fig 4 present the model fitting results (p<0.05 for the fitted *β*_1_ *and β*_2_ values). As an exploratory analysis, we added the population of each destination country as an explanatory variable; however, the population variable is tested insignificant.

**Table 3:** Parameters of Gravity Model Fitting.

*Country*	*β*_*1*_	*β*_*2*_	*Goodness of Fit*
***United States***	0.888907	0.852754	0.696901
***United Kingdom***	0.282591	0.723032	0.888197
***Spain***	0.663266	0.725101	0.836836
***Germany***	0.299241	0.520118	0.702163
***Italy***	0.537103	0.820647	0.865106
***France***	0.278054	0.557594	0.823368
***Canada***	1.146631	0.494434	0.937137
***Australia***	0.029144	0.927834	0.838571
***Brazil***	1.049459	0.429203	0.721705
***Japan***	0.260822	0.877435	0.868886
***Netherlands***	0.574255	0.751652	0.83742
***China***	0.857574	0.791574	0.792836

**Fig 5:**
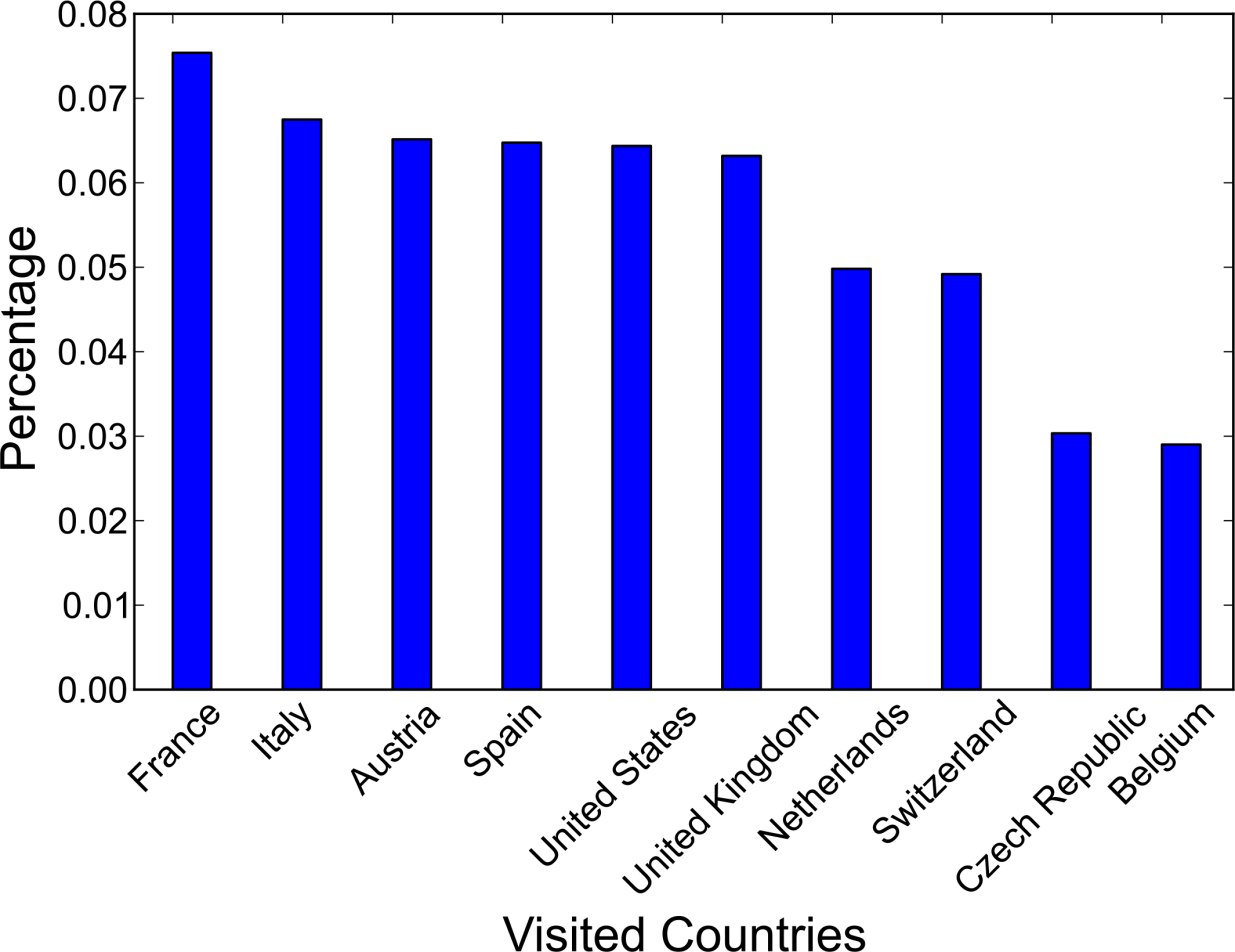
Visiting Patterns (Germany).

As can be seen, the 12 selected countries exhibit distinct patterns regarding the fitted *β* values. Descriptively, we can observe the following four patterns (note that here small and large *β* values are defined as a value lower/higher than the median of the selected sample set):

- Countries with a small *β*_1_ value but a large *β*_2_ value–international travel destinations are more influenced by the popularity of destination instead of the distance from the origin. One example is Australia, which is more “geographically isolated” from the other continents and naturally the residents may need to travel far away to reach a foreign destination. This “geographical isolation” results in long international travels (the top two countries Australians travel to are the U.S. and the U.K., whereas a much closer destination, New Zealand, only comes in third place).
- Countries with a large *β*_1_ value and a large *β*_2_ value–international travel destinations are influenced by both the popularity of the destination and the distance from the origin. One example is the U.S., where residents show mixed interest in both adjacent destinations (e.g., Canada) and popular but faraway destinations, such as the U.K. (Fig 2).
- Countries with a large *β*_1_ value but a small *β*_2_ value–international travel destinations are mainly influenced by the distance between the origin and the destination countries. One example is Canada, where over 25% of the travel destinations from Flickr users are to the U.S.
- Countries with a small *β*_1_ value and a small *β*_2_ value–international travel destinations are not substantially influenced by the popularity of the destination or distance. One example is Germany, where destination choices are more evenly distributed and do not exhibit clear patterns (Fig 5).

Compared to a similar work by Hawelka et al. [24], the gravity models in this research are fitted for each country, and we identified four categories of countries based on the fitted *β* values (countries with a large *β*_1_ value but a small *β*_2_ value, countries with a large *β*_1_ value and a large *β*_2_ value, countries with a small *β*_1_ value but a large *β*_2_ value, and countries with a small *β*_1_ value and a small *β*_2_ value), whereas in [24], the authors fit one gravity model for all countries at the global level. Compared to a unified conclusion that “people tend to travel to close-by destinations,” our work reveals a categorical pattern for different countries.

Fig 4 also indicates interesting regional patterns regarding international traveling for countries. For instance, the five European countries (Germany, Netherlands, Spain, Italy, and France) locate close to the diagonal line, indicating that both *β*_1_ and *β*_2_ play an equal role in determining the travel destinations in those countries. One exception is the U.K., where the distance plays a weaker role than the popularity of the destination countries. This is potentially due to the relatively “isolated” geographic location of the U.K. compared to the other European countries in this study. Another interesting finding is regarding countries with similar cultural backgrounds. For instance, both China and Japan are East Asian countries and share a considerable cultural history. For instance, both are considered high-context cultures, in which context plays a crucial role in communicating complex messages effectively [53, 54]. However, the users in these two countries exhibit very different patterns in the choice of travel destinations. Even though both countries show large ROG values, for Japanese users the impact of distance on international traveling is even weaker, instead, they prefer to go to popular travel destinations regardless of distance (i.e., the top two destinations are the U.S. and the U.K.). A noticeable difference also exists for U.S. and Canadian users, where distance plays a more important role for Canadian users than the attraction of the destinations. However, both *β*_1_ and *β*_2_ play an equally important role for U.S. users.

### 4.3. Discussion of Uncertainty

It is also important to highlight the different aspects of uncertainty related to human activity studies in this research. These issues arise in our data mining process in different ways [3, 55], including but not limited to:

- Natural variability of human mobility: Although human activities seem to be highly predictable [4, 44], randomness is an inevitable part of human motion.
- Inaccuracy/imprecision due to the limitation of available data: Positional inaccuracy, sampling resolution, and imprecision all contribute to the uncertainty of our data source. First, the accuracy of positioning data often depends on the GPS signal level in the study area. Second, the location records in the dataset cannot represent the accurate travel trajectories of each user, since the locations are recorded only when a geotagged photo is uploaded. Third, the precision of spatial information varies for different datasets, e.g., a record such as “126.51551E, 45.15153N” is more precise than “126.52E, 45.15N.”

**Fig 6:**
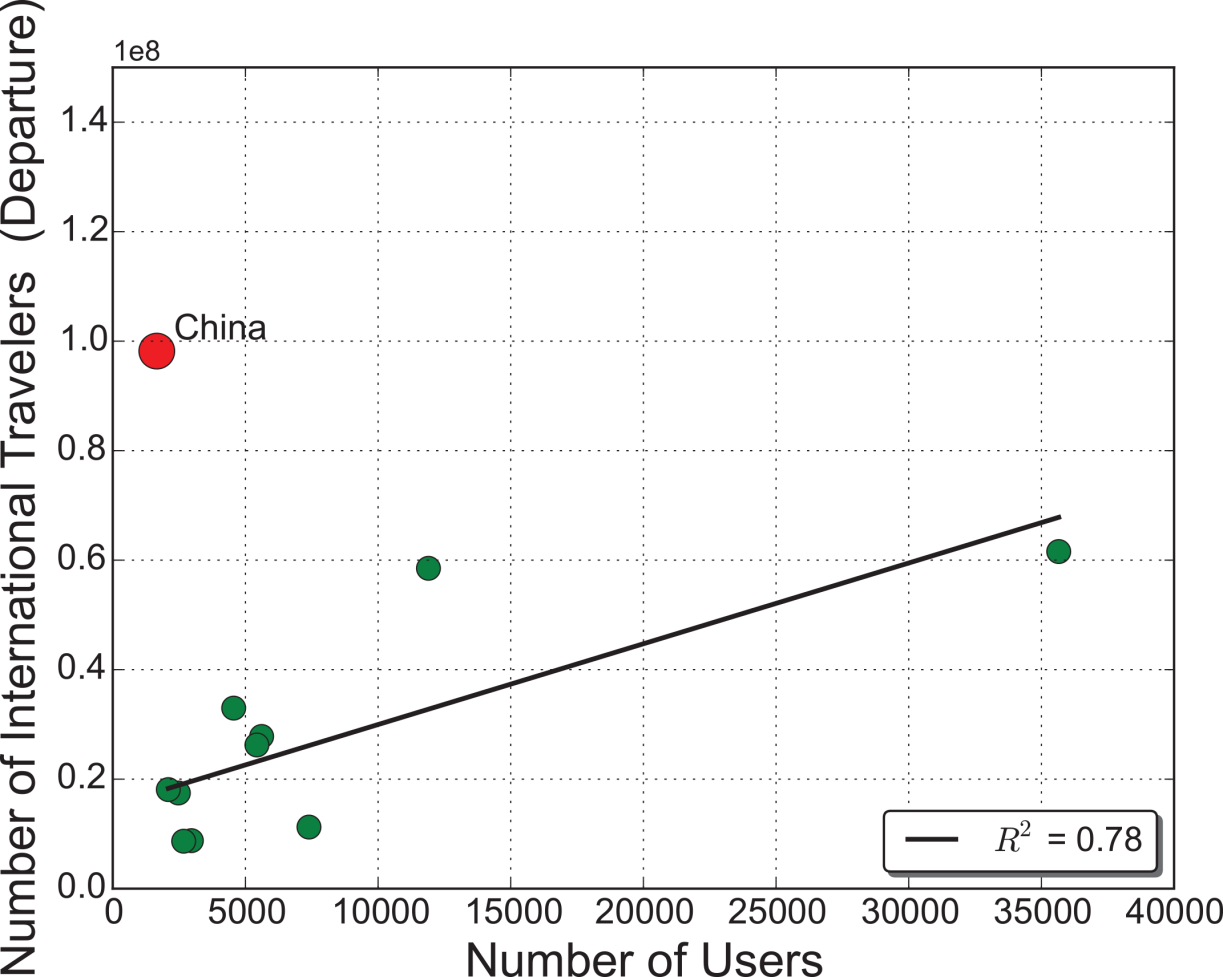
Correlation between the number of Flickr users and official travel statistics.

Additionally, to investigate the sampling bias of Flickr, we also calculate the correlation coefficient between the number of Flickr users and the official travel statistics published by the World Bank (i.e., number of international travelers departing from each country) in the top 12 countries (Germany is excluded because the data is not provided by the World Bank). From the data we can observe a substantial discrepancy between the amount of Chinese users on Flickr and the actual number of international travelers departing from China (Fig 6). This is potentially due to the fact that Flickr is mainly used in North America and Europe, and it is not particularly popular among Chinese users. For the remaining ten countries, the correlation between the number of tourist departures and the number of users on Flickr is 0.78. This on the one hand helps justify the choice of using Flickr to explore international traveling, and on the other hand, it reveals the sampling bias and potential insufficiency of LBSM for certain geographic regions such as China.

- Imperfection of models and algorithms: As Box and Draper [56] (pp.424) stated: “Essentially, all models are wrong, but some are useful.” In this research, a variation of the gravity model of trade is adopted to interpret the interaction of travel behavior, due to the fact that it is a flexible model to reflect directional interaction of international travel flow. However, other models may be applicable, such as Ullman's spatial interaction model [57]. The application of different models will inevitably have an impact on the uncertainty of the results. In future studies it will be helpful to validate the results with other data sources to test their robustness.

## 5. Conclusion

The relationship between LBSM data and human activity patterns has been widely studied in various fields such as transportation, computer science, urban planning, and computational physics. Such data, as an input to the analysis of human mobility, has the potential to transform research in diverse fields, such as geography, transportation, planning, and economics. This research explored international travel patterns of Flickr users. The major contributions of this research are:

1. We applied three measurements to explore descriptive statistics of travel flows between countries. The results indicate that travel distance (ROG) is highly influenced by how geographically “isolated” a country is as well as the size of the home country, and the number of countries visited exhibit a relatively stable pattern, i.e., the average number of countries visited is between 3–4 in the majority of the 12 selected countries. The entropy values indicate that the travel interests of European residents are more evenly spread based on the number of photos uploaded at each destination.
2. We explored the potential of applying a variation of the gravity model of trade to analyze outgoing travel flow in different countries. Four types of patterns were observed based on how *the popularity of a destination country* and *the distance between the destination and origin countries* affect travel choices. We also observed interesting patterns of distinct travel choices among countries with similar cultural backgrounds.

The results of this research provide valuable input in quantifying international travel patterns in the age of instant access. Analyzing the travel behavior of LBSM users offers quantitative support to many commercial applications, such as individualized searching and advertising, tourism sustainable planning, or hotspot identification. This research can be extended from several perspectives. The methods and models can be applied to other LBSM datasets (e.g., Twitter or Foursquare) to test their robustness. Geotagged photos also provide a rich data source to analyze inter-region travel flows at various spatial scales, such as investigating the connection between different provinces in China. Also, further research may involve comparing social media and traditional survey data in an effort to characterize urban-level patterns. Future studies can also look into the correlation between inter-region interactions and various demographic variables (i.e., how the socio-economics of the built environment may play a role in such interaction). The three measurements can also be incorporated into predictive models for individual travel planning and destination suggestions.
